# Effect of Urban Particulate Matter on Vocal Fold Fibrosis through the MAPK/NF-κB Signaling Pathway

**DOI:** 10.3390/ijms21186643

**Published:** 2020-09-10

**Authors:** Ho-Ryun Won, Seung-Nam Jung, Min-Kyung Yeo, Shinae Yi, Lihua Liu, Mi Ae Lim, Chan Oh, Yea Eun Kang, Jae Won Chang, Ki Sang Rha, Bon Seok Koo

**Affiliations:** 1 Department of Otolaryngology-Head and Neck Surgery, College of Medicine, Chungnam National University, Daejeon 35015, Korea; hryun83@gmail.com (H.-R.W.); jasick@hanmail.net (S.-N.J.); dlaaldo22@naver.com (M.A.L.); strive1005@daum.net (J.W.C.); 2 Department of Pathology, College of Medicine, Chungnam National University, Daejeon 35015, Korea; mkyeo83@cnuh.co.kr; 3 Department of Endocrinology and Metabolism, College of Medicine, Chungnam National University, Daejeon 35015, Korea; kikirabbit21@naver.com (S.Y.); yeeuni220@naver.com (Y.E.K.); 4 Department of Medical Science, College of Medicine, Chungnam National University, Daejeon 35015, Korea; 18744308309@163.com (L.L.); ohchanny@naver.com (C.O.)

**Keywords:** urban particulate matter, vocal fold fibroblast, vocal fold fibrosis, laryngitis, voice disorder

## Abstract

Particulate matter (PM) is an environmental exposure factor that adversely affects human health. PM is a risk factor for various diseases. However, the mechanism by which PM affects the vocal folds (VF) has not yet been evaluated. Thus, we investigated the cytotoxic effects of PM on human vocal fold fibroblasts (hVFF) and the underlying signaling pathways. hVFF were isolated from human VF. The effect of PM on hVFF, and the underlying mechanism, were analyzed using Western blot, quantitative real-time polymerase chain reaction, and flow cytometry. In addition, a histological evaluation was performed in animal experiments. Cell proliferation decreased after the PM treatment. PM increased the expression of interleukin (IL)-6 and IL-1β. The generation of reactive oxygen species (ROS) in PM-treated hVFF and subsequent activation of the mitogen-activated protein kinase (MAPK) and nuclear factor-κB (NF-κB) pathways were confirmed. Furthermore, PM increased the expression of fibrosis-related markers and induced the accumulation of collagen in the extracellular matrix. As a result, PM exposure significantly enhances the inflammatory response on VF through the ROS-mediated activation of the MAPK and NF-κB signaling pathways. In addition, PM promotes differentiation into myofibroblasts and induces fibrosis. These results suggest that PM triggers an inflammatory reaction through ROS production and causes VF fibrosis.

## 1. Introduction

Airborne particulate matter (PM) causes many environmental and social problems globally. According to recent reports, air pollution is estimated to contribute to the deaths of approximately 1.3 million people worldwide each year [1], and PM is one of the leading causes of air pollution. Several epidemiological studies have shown that PM can lead to the exacerbation of diseases related to the respiratory tract, such as asthma, lung cancer, and chronic obstructive pulmonary disease [2,3,4,5]. PM affects different body parts depending on the particle diameter; for example, PM 10 (≤10 μm) affects the upper respiratory tract, including the nasal cavity and bronchial epithelium [6,7]. On the other hand, PM 2.5 (≤2.5 μm) passes through the upper respiratory tract, is deposited, and affects the entire airway, especially small airways and alveoli [8]. The larynx and vocal folds (VF) are important parts of the upper respiratory tract, but no studies have explored their response to PM.

Voice disorders are common in the United States, affecting 7–8% of the population each year [9,10], and with a direct healthcare cost estimated to be up to $5 billion annually [11]. Human vocal fold fibroblasts (hVFF) are important cells that determine the composition of the extracellular matrix, such as collagen deposition in the VF body, among several cells constituting VF, such as epithelial cells, endothelial cells, and myocytes [12]. VF fibrosis is often caused by the healing process that occurs after the inflammation of the VF [13]. During VF inflammation, hVFF distributed in the viscoelastic layer and the edge of the VF differentiates into myofibroblasts, which increases collagen accumulation and thus increases the stiffness of the vibrating part of the structure [14]. Therefore, the inflammation of the VF is a major cause of voice disorders.

In this study, we evaluated the effect of PM on hVFF and identified the mechanism underlying the cytotoxic effect of PM on the hVFF. We also sought to determine whether PM-induced inflammation directly promotes VF fibrosis.

## 2. Results

### 2.1. Correlation between Airborne PM 10/2.5 Concentration and Acute Laryngitis/Tracheitis

First, under the hypothesis that PM may cause an inflammatory reaction in the VF, a simple statistical comparison was conducted to determine if there was a correlation between the concentration of PM and the prevalence of upper respiratory tract infections (laryngitis/tracheitis). From January 2016 to December 2018, the average PM concentration in the air and the number of laryngitis/tracheitis cases in Seoul, Republic of Korea, were analyzed. PM is classified into PM 10 and PM 2.5 depending on the particle diameter. It was confirmed that the number of patients with acute laryngitis/tracheitis increased as the concentration of PM 10 and PM 2.5 increased, and vice versa (Figure S1A). Both the PM 10 (r = 0.593, *p* < 0.001) and PM 2.5 (r = 0.533, *p* < 0.001) concentrations showed a significant positive correlation with the incidence of acute laryngitis/tracheitis (Figure S1B). Based on these results, the concentration of airborne PM is related to inflammation in the larynx and VF.

### 2.2. PM Reduces the Viability of hVFF

**Figure 1:**
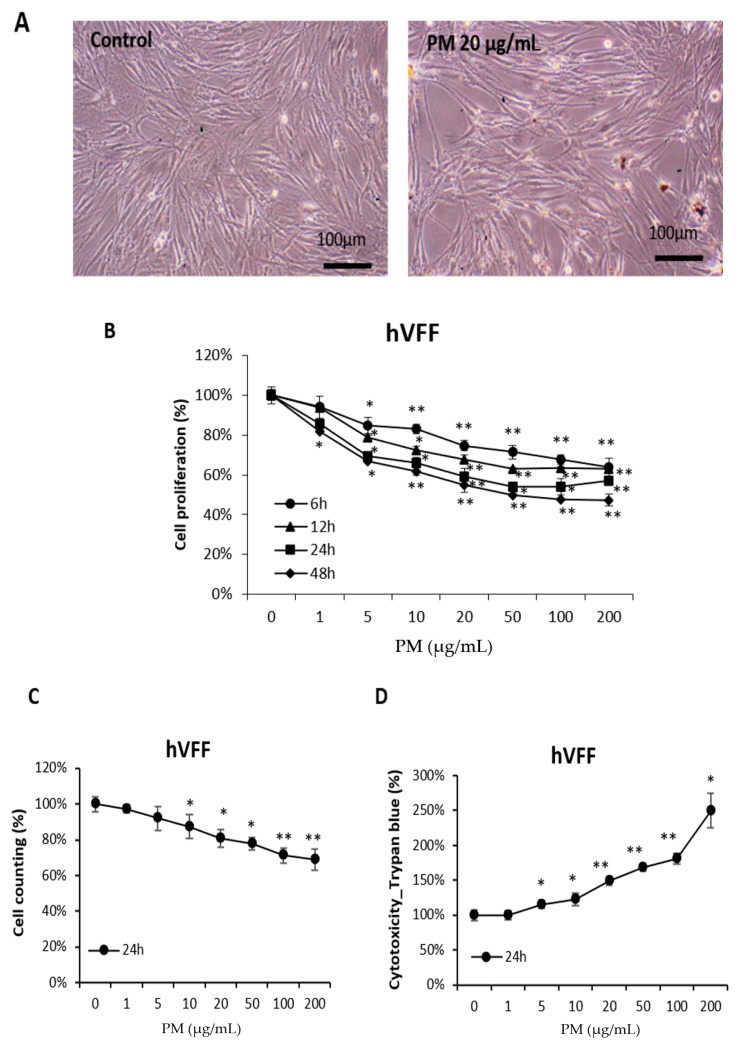
Effects of PM on the viability of hVFF. (**A**) Microscopic evaluation of cell morphology. Cells were treated with PM (20 μg/mL) for 12 h. (**B**) Results of cell proliferation assays. Cells were treated with various concentrations of PM (0–200 μg/mL) for 6, 12, 24, and 48 h. (**C**) Results of cell counting assays. Cells were treated with various concentrations of PM (0–200 μg/mL) for 24 h. (**D**) Results of the cell cytotoxic assays. Cells were treated with various concentrations of PM (0–200 μg/mL) for 24 h. PM induces cytotoxicity in hVFF and decreases cell proliferation. * *p* < 0.05, ** *p* < 0.01.

Based on the results shown in Figure S1, the cell proliferation after treating normal hVFF with PM was assessed to determine the effect of PM on VF. Optical microscopy showed that the number of hVFF decreased after PM treatment (Figure 1A). To quantify this finding, a cell proliferation assay (Figure 1B) and cell counting assay (Figure 1C) were performed. As the concentration of PM increased, the cell proliferation significantly decreased, similar to the effect of the increased PM treatment time. In addition, a cell cytotoxic assay was performed, and it was confirmed that the cell cytotoxicity increased significantly as the concentration of PM treatment increased (Figure 1D). These results suggest that PM is cytotoxic to hVFF to a degree proportional to the concentration and treatment time.

### 2.3. PM Induces Pro-Inflammatory Cytokines in hVFF through Nuclear Factor-κB (NF-κB) Signaling

**Figure 2:**
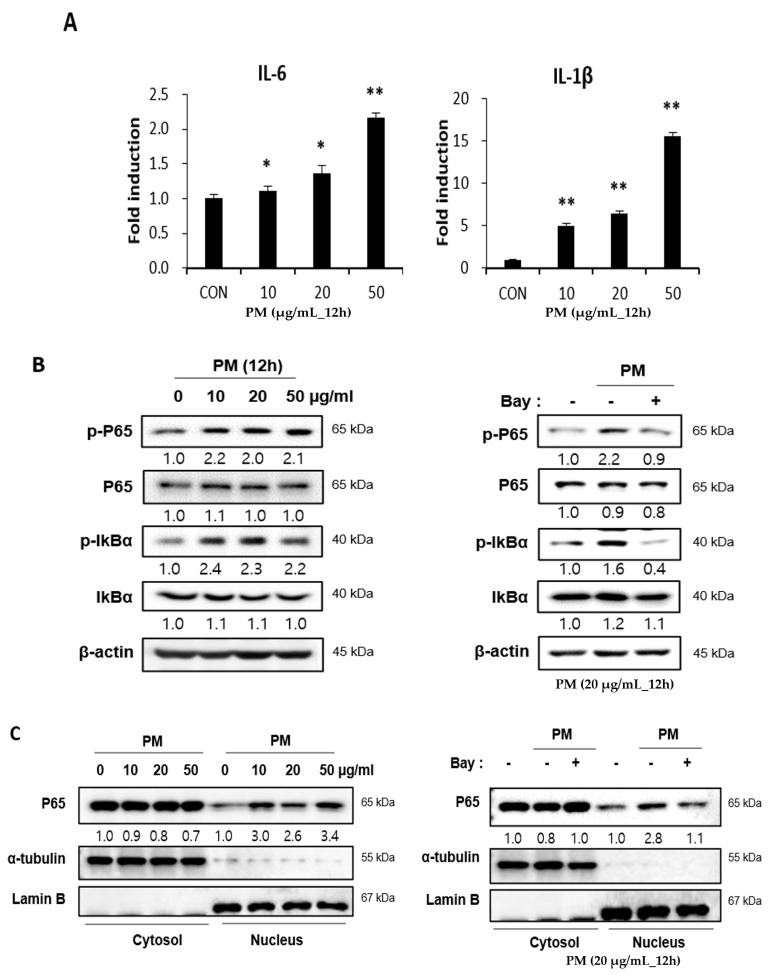

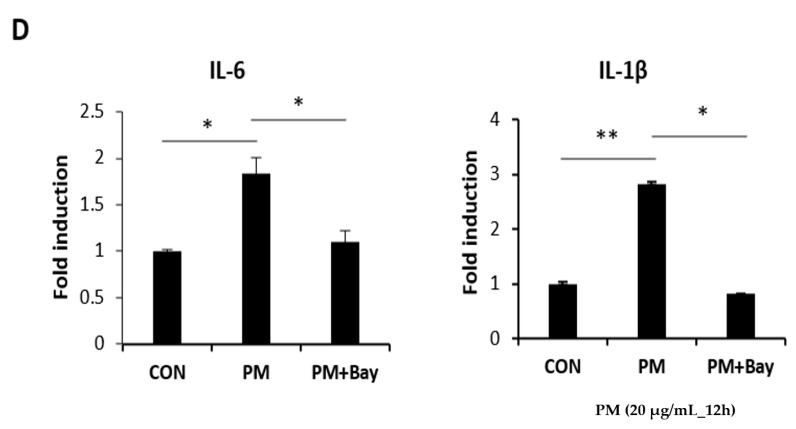
PM induces pro-inflammatory cytokines through the NF-κB signaling pathway. (**A**) The mRNA levels of IL-6 and IL-1β were verified by RT-PCR. PM increased the mRNA expression of IL-6 and IL-1β. (**B**) The expression of proteins associated with the NF-κB signaling pathway was evaluated by Western blot. The expression of p-P65 and p-IκBα increased significantly with the PM treatment. Cells were treated with various concentrations of PM (0–50 μg/mL) for 12 h. Increased expression of the protein associated with NF-κB signaling pathway activation was reduced by NF-κB inhibition (Bay 11-7082). (**C**) The expression of P65 in the cytoplasm and the nucleus. The translocation from the cytoplasm to the nucleus was increased after the PM treatment. Cells were treated with various concentrations of PM (0–50 μg/mL) for 12 h. The translocation from the cytoplasm to the nucleus was reduced by NF-κB inhibition. (**D**) The increased mRNA expression of IL-6 and IL-1β was reduced significantly by NF-κB inhibition (Bay 11-7082). Cells were treated with PM (20 μg/mL) for 12 h. * *p* < 0.05, ** *p* < 0.01.

PM induces pro-inflammatory cytokines in cells of the airway mucosa, such as nasal fibroblasts and bronchial epithelial cells, which have been reported to be expressed following the activation of the NF-κB signaling pathway [6,7,15]. Therefore, the expression of pro-inflammatory cytokines after PM treatment on hVFF was assessed at the messenger RNA (mRNA) level. As the concentration of PM increased, the expression of pro-inflammatory cytokines, such as interleukin (IL)-6 and IL-1β, increased significantly (Figure 2A). Next, to confirm activation of the NF-κB signaling pathway by PM, the expression levels of the total P65 and phospho(p)-P65 proteins, which are involved in NF-κB heterodimer formation, were measured. There was no change in the expression of total P65 after the PM treatment (Figure 2B). Therefore, the expression of P65 in the cytoplasm and nucleus was confirmed. After the PM treatment, it was confirmed that translocation from the cytoplasm to the nucleus was increased (Figure 2C), and this was confirmed to be reduced by the NF-κB inhibitor (Bay 11-7082) (Figure 2C). In addition, the p-P65 expression increased after the PM treatment (Figure 2B). Nuclear factor of kappa light polypeptide gene enhancer in B-cells inhibitor alpha (IκBα) inhibits the expression of the NF-κB transcription factor; NF-κB is activated by the phosphorylation of IκBα [16]. After PM treatment, p-IκBα showed increased expression (Figure 2B). Additionally, when treated with an NF-κB inhibitor, the increases in p-P65 and p-IκB induced by PM were reversed (Figure 2B), and the concentration of pro-inflammatory cytokines decreased (Figure 2D). Thus, PM causes inflammatory responses in hVFF by increasing the expression of pro-inflammatory cytokines through NF-κB signaling.

### 2.4. PM Induces NF-κB Signaling through the Mitogen-actiVated Protein Kinase (MAPK) Pathway

**Figure 3:**
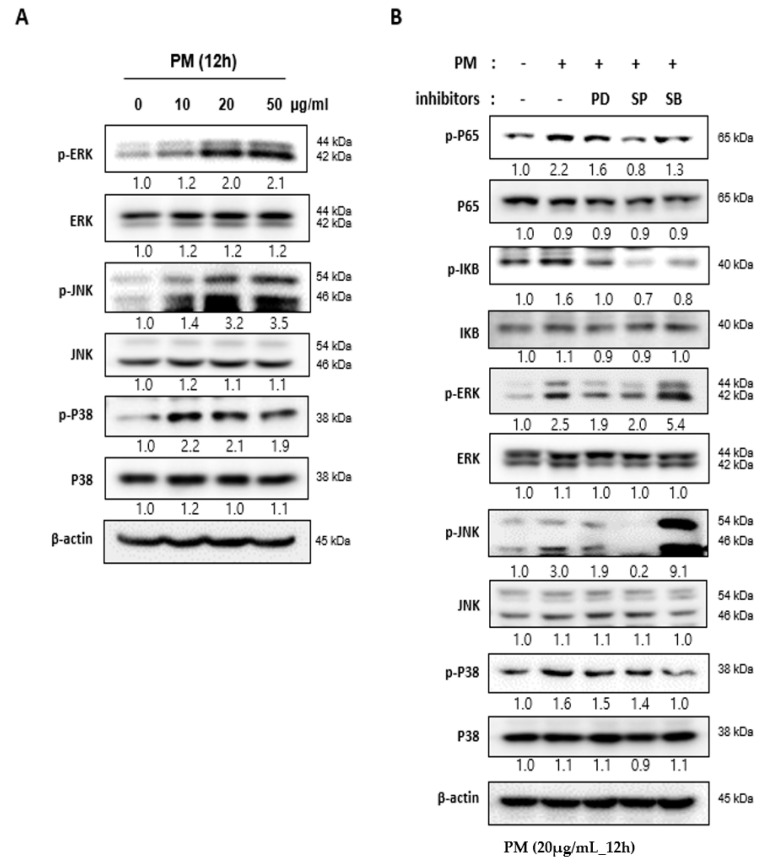
PM induces activity in the NF-κB signaling pathway through the MAPK pathway. (**A**) The expression levels of the MAPK family members ERK, JNK, and P38 were evaluated by Western blot. The levels of p-ERK, p-JNK, and p-P38 expression increased according to the PM concentration. Cells were treated with various concentrations of PM (0–50 μg/mL) for 12 h. (**B**) Increased expression of the protein associated with the activation of the MAPK signaling pathway was reduced by specific inhibitors (ERK inhibitor: PD98059; JNK inhibitor: SP600125; P38 inhibitor: SB203580). The increased expression of p-P65 after the PM treatment was also reduced by the inhibitors. Cells were treated with PM (20 μg/mL) for 12 h.

The MAPK family is a group of signaling mediators involved in the regulation of inflammatory responses [17]. Three important MAPK pathways are the extracellular signal-regulated kinase (ERK), c-Jun N-terminal kinase (JNK), and the P38 MAPK pathways, which regulate NF-κB signaling [15,18]. When hVFF was treated with PM, the expression levels of p-ERK, p-JNK, and p-P38 increased in proportion to the concentration (Figure 3A), and the p-P65 expression decreased after treatment with MAPK pathway inhibitors (ERK inhibitor: PD98059; JNK inhibitor: SP600125; P38 inhibitor: SB203580) (Figure 3B). These results suggest that the inflammatory response induced by PM in hVFF is regulated by the MAPK/NF-κB signaling pathway.

### 2.5. Reactive Oxygen Species (ROS) Induced by PM in hVFF Activate the MAPK Pathway

**Figure 4:**
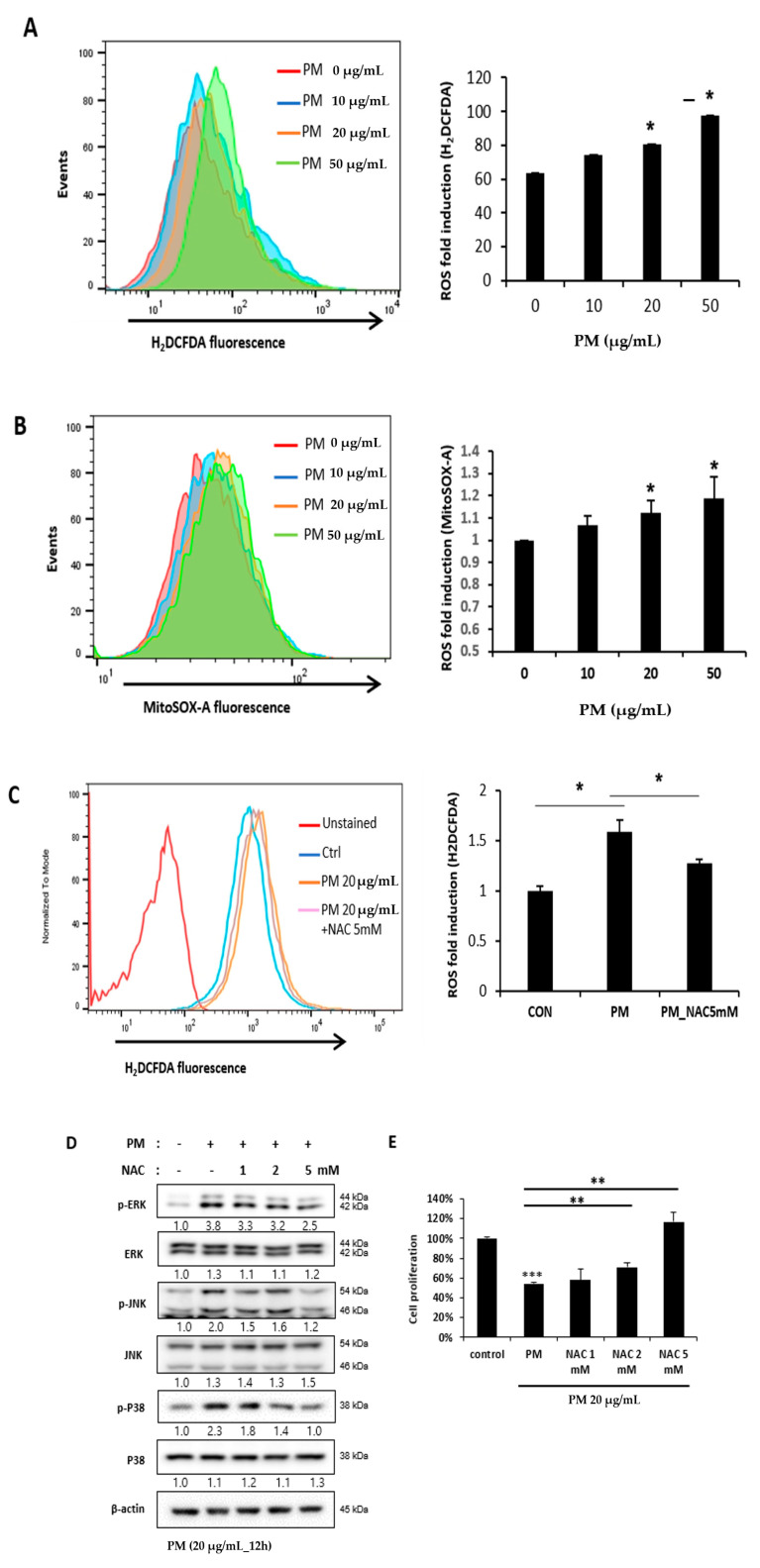
PM-induced ROS activates the MAPK pathway. (**A**,**B**) Cytoplasmic ROS and mitochondrial ROS expression after the PM treatment was evaluated. Both the cytoplasmic and mitochondrial ROS increased significantly with the PM concentration. Cells were treated with various concentrations of PM (0–50 μg/mL) for 12 h. (**C**) The increased ROS expression was decreased significantly by the ROS scavenger NAC (5 mM). (**D**) The increased expression of the protein associated with the activation of the MAPK signaling pathway was reduced by NAC (5 mM). (**E**) Cell proliferation, which was reduced after the PM treatment, recovered significantly in proportion to the concentration of NAC (0–5 mM). Cells were treated with PM (20 μg/mL) for 12 h. * *p* < 0.05, ** *p* < 0.01, *** *p* < 0.001.

ROS is a chemically reactive species containing oxygen known to cause oxidative stress. ROS cause many diseases and conditions associated with PM, including inflammation, and trigger the activation of the MAPK pathway [15,19]. After PM treatment, it was confirmed that the expression of both the cytoplasmic and mitochondrial ROS of hVFF increased significantly with the increased PM concentration (Figure 4A,B). N-acetylcysteine (NAC), an aminothiol and precursor to glutathione, is a scavenger with an antioxidant effect that inhibits ROS [20]. We confirmed that the increase in ROS induced by PM was significantly decreased by NAC (Figure 4C). In addition, the MAPK pathway activation by PM was inhibited by NAC (Figure 4D). The cell proliferation, which decreased after the PM treatment, significantly recovered in proportion to the concentration of NAC (Figure 4E). Taken together, the results showed that PM activates the MAPK pathway by increasing the ROS in hVFF. Through NF-κB signaling, PM promotes the expression of pro-inflammatory cytokines and the inflammatory response, and decreases cell viability.

### 2.6. PM Induces the Differentiation of hVFF into Myofibroblasts

**Figure 5:**
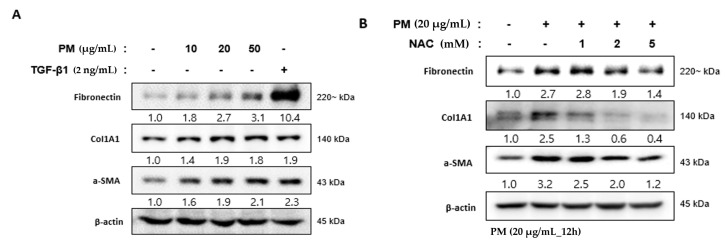
PM-induced differentiation of hVFF into myofibroblasts. (**A**) Fibrosis-related molecules were measured by Western blot. Fibronectin, ColA1, and α-SMA increased after the PM treatment, similar to after the TGF-β1 treatment (2 ng/mL). Cells were treated with various concentrations of PM (0–50 μg/mL) for 12 h. (**B**) The increased expression of fibronectin, ColA1, and α-SMA was reduced by NAC (0–5 mM). Cells were treated with PM (20 μg/mL) for 12 h.

hVFF constituting the VF mucosa differentiate into myofibroblasts after damage or inflammation, which leads to excessive collagen accumulation in the extracellular matrix (ECM) [14]. This excessive accumulation of collagen is an important mechanism of fibrosis of the VF mucosa [21,22]. Myofibroblast differentiation can be induced intentionally by treatment with transforming growth factor β1 (TGF- β1) [23]. After the TGF-β1 treatment of hVFF, fibrosis-related markers such as fibronectin, α-smooth muscle actin (α-SMA), and alpha-1 type I collagen (Col1A1) increased at the protein level, similar to after PM treatment (Figure 5A). In addition, the fibrosis-related markers upregulated by PM returned to normal levels after the NAC treatment (Figure 5B). These results suggest that PM promotes the differentiation of hVFF into myofibroblasts due to the inflammatory reaction induced by the development of ROS, which in turn induces fibrosis of the VF mucosa.

### 2.7. PM Induces Vocal Fold Mucosal Inflammation and Modulates ECM Deposition In Vivo

**Figure 6:**
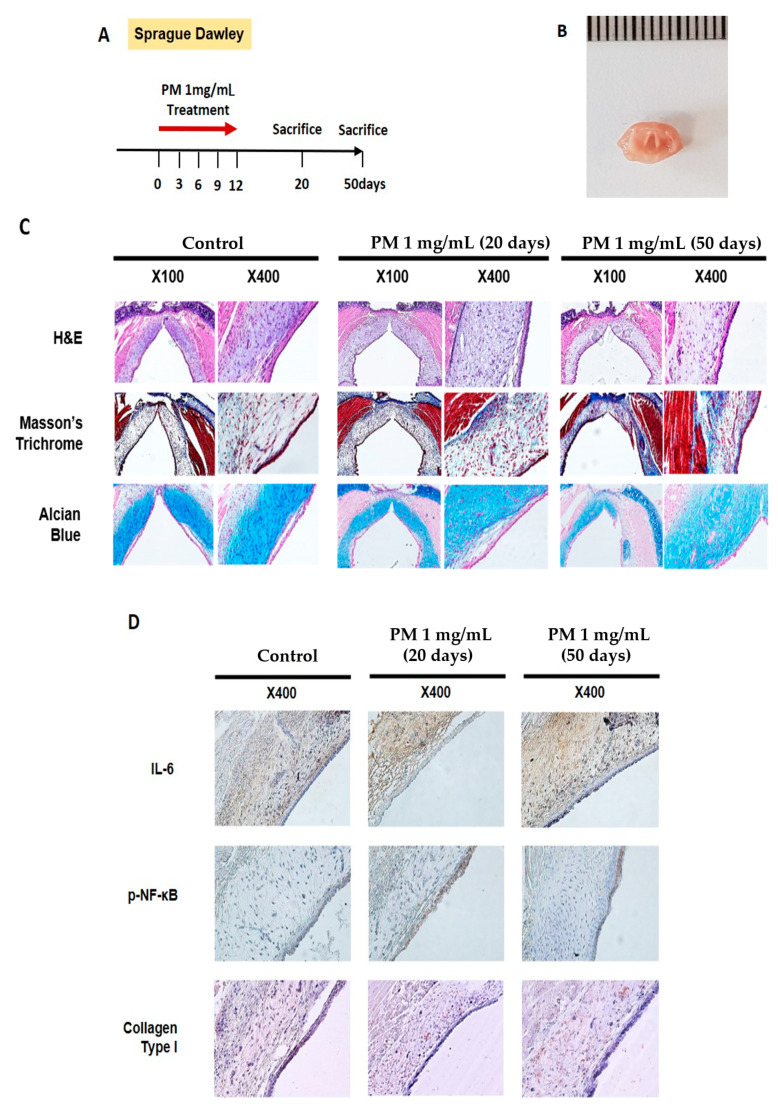

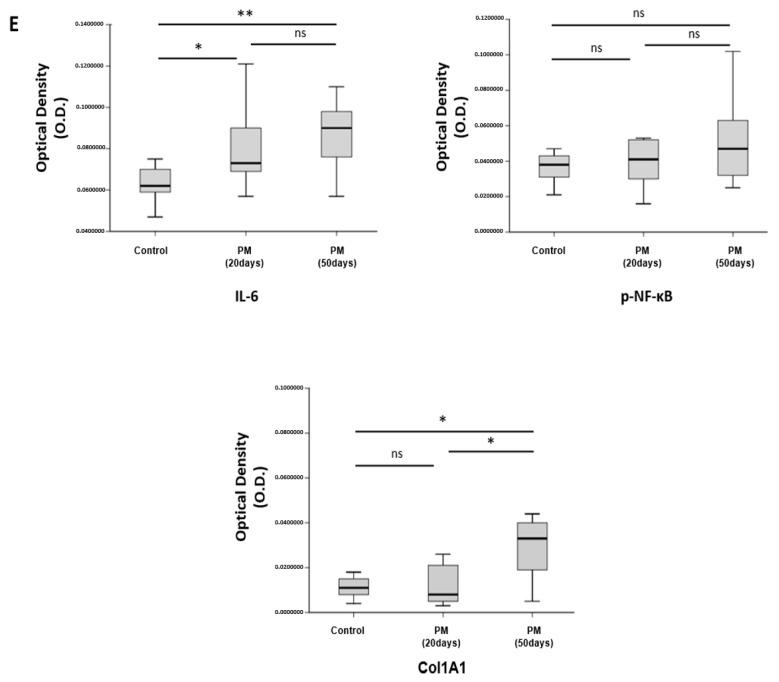
PM induces VF mucosal inflammation and modulates ECM in vivo. (**A**) Study protocol of the experimental group. In the experimental group, 1 mL of PM diluted in 1 mg/mL distilled water was administered through the oral cavity a total of five times every per 3 days. (**B**) Gross morphology of VF excised from sacrificed animals. (**C**) Histological analysis of VF in the control group 20 and 50 days after PM treatment. H&E staining showed that the thickness of the lamina propria was increased on day 20. VF in the PM-treated group was characterized by an irregularly arranged lamina propria on day 50 (upper-panel). In Masson’s trichrome staining, collagen was dyed dark blue. Collagen deposition was increased in the experimental group compared to the control group (mid-panel). Alcian blue stains hyaluronic acid blue. The amount of hyaluronic acid decreased slightly in the PM-treated group (bottom-panel). (**D**) Immunohistochemical analysis of VF after normal saline and PM treatment. High expression of IL-6 and collagen type I in the lamina propria was observed in the experimental group. The expression of p-NF-κB was also increased in the epithelium after the PM treatment. (**E**) Quantitative analysis results for immunohistochemical analysis. * *p* < 0.05, ** *p* < 0.01, ns; non-specific.

Animal experiments were performed to evaluate the effect of PM on VF in vivo. PM (1 mg/mL, 1 mL) was applied once every per 3 days using a throat sprayer for a total of 5 applications, and was evaluated by sacrifice on days 20 and 50 (Figure 6A). After the sacrifice, only the glottic lesion was dissected (Figure 6B), after which histologic evaluation was performed. Hematoxylin and eosin (H&E) staining on day 20 confirmed that the lamina propria was thicker, and the aggregation of cells that appeared to be inflammatory increased compared to the control group. On day 50, the VF in the PM-treated group were characterized by an irregularly arranged lamina propria (Figure 6C, upper-panel) With Masson’s trichrome staining, collagen is dyed dark blue and can thus be characterized. The collagen deposition in the lamina propria on day 20 after PM treatment was not significantly different from the control group, but was greater on day 50 (Figure 6C, mid-panel). Alcian blue stains hyaluronic acid blue. After PM treatment, the deposition of hyaluronic acid was somewhat reduced compared to the control group on days 20 and 50 (Figure 6C, bottom-panel).

Immunohistochemical analysis showed statistically increased IL-6 in the lamina propria in the group treated with PM (Figure 6D, upper-panel, and Figure 6E). The expression of p-NF-κB was not statistically significant in the PM treatment. However, it was identified an increasing pattern mainly in the epithelium (Figure 6D, mid-panel, and Figure 6E). The deposition of Col1A1 in the lamina propria did not show any specific change on day 20 after the PM treatment; however, the deposition was noticeably increased on day 50 (Figure 6D, bottom-panel, and Figure 6E).

## 3. Discussion

In this study, PM was cytotoxic to hVFF, and was associated with the increased expression of pro-inflammatory cytokines such as IL-6 and IL-1β. It was confirmed that pro-inflammatory cytokines are mediated by the MAPK/NF-κB signaling pathway, and PM-induced ROS generation is known to increase the activity of these pathways. In addition, the inflammatory response induced by PM increased the rate of cellular differentiation into myofibroblasts, suggesting that it may accelerate VF fibrosis.

As one of the major air pollutants, PM is a complex mixture of different-sized particles varying in origin and chemical composition [6]. Dust, smoke, and soot are among the various types of PM, and are emitted directly into the air or generated by gas pollutants. PM of various origins is known to be associated with the development or exacerbation of various diseases such as bronchial asthma, chronic obstructive pulmonary disease, lung cancer, pulmonary fibrosis, and cardiovascular disease [2,3,5,24,25,26,27]. Recently, PM 0.1 (≤0.1 μm), which is classified as ultrafine PM, was shown to be absorbed not only through the respiratory system, but through the circulatory system, and has been reported to affect individuals with obesity, diabetes, neurodegenerative diseases, and congenital defects [28,29,30]. However, studies on the effects of PM on the upper respiratory tract are limited compared to those on the lower respiratory tract [6,31]; this is the first study to analyze the effects of urban PM on the larynx, especially the VF.

PM is either accumulated or absorbed, and affects the body through various signaling pathways. Cell death, such as apoptosis [18], and the epithelial-to-mesenchymal transition are regulated through complex pathways [32,33]. However, the most common pathological etiology is the inflammatory response, which is induced through various pathways depending on the metal and organic components of PM, and can be classified as oxidant or non-oxidant-mediated [15]. Oxidative stress induced by PM, a direct increase in ROS [34,35,36], or interactions with the cellular membrane and receptors on the cell surface can induce an inflammatory response [37,38]. The most commonly activated signaling pathways are the MAPK and NF-κB pathways [17,39].

NF-κB is a transcription factor that plays a critical role in inflammation and in cell immunity, proliferation, and apoptosis [16]. NF-κB signaling is regulated through the MAPK signaling pathway, which is one of the most important intracellular signal-transduction pathways [17]. Wang et al. reported that PM-induced lung inflammation was caused by the ROS-MAPK-NF-κB signaling pathway. Furthermore, it has been reported that the activation of the MAPK/NF-κB pathway is associated with the hyperreactivity or inflammation of the airway epithelium by PM, and also affects the endothelial cell barrier [8,40,41]. The mechanism by which PM affects nasal fibroblasts has recently been characterized [6]. We hypothesized that hVFF, as components of the VF, which is part of the upper airway directly affected by PM, will be affected through the same mechanism. We confirmed for the first time that PM directly increases inflammation in the hVFF through the ROS-induced activation of the MAPK/NF-κB signaling pathway.

Voice disorders affect 7–8% of adults in the United States each year, and significantly impact quality of life [9,10]. Voice disorders are caused by the improper contraction of the VF muscles, deformation of the edges of the VF, thinning of the viscoelastic layer of the lamina propria, or the stiffness of the vibrating part of the VF. These changes are mainly caused by inflammatory reactions to surgery or infection [42,43]. When the inflammatory response is induced, hVFF differentiate into myofibroblasts by signal transduction [14]. Myofibroblasts alter the composition of the ECM through excessive collagen accumulation, thereby increasing the stiffness of the VF, which in turn causes voice disorders [21]. We explored whether acute laryngitis/tracheitis was significantly correlated with the concentration of PM in the air by analyzing epidemiological data prior to the experiment. In vitro experiments confirmed that such inflammation can be directly induced by PM and may lead to VF fibrosis. These results were supported by animal experiments. Of course, the probability of a voice disorder cannot be determined simply based on in vitro experiments and histological findings. In addition, the number of animal experiments performed in this study is too small. Since rodents were used as an experiment, there is a limit to collecting only microscopic vocal fold tissue, and this limitation is thought to have some influence on the results. Therefore, a physiological assessment of the voice through additional animal experiments is required. Nevertheless, this study has important implications, suggesting that PM causes voice disorders through the direct inflammation of the VF.

PM is often classified according to size, because the effects on the human body depend on the aerodynamic diameter of the particles. PM 10 deposits mainly in upper respiratory tract areas, such as the nasal cavity, while PM 2.5 accumulates in small airways such as alveoli. PM 0.1 is absorbed by the alveoli and can reach the pulmonary circulation [2,44]. Urban PM is composed of solid and liquid particles with various chemical components. As well as particle size, inflammation may also depend on the chemical composition of PM [8,45,46]. The urban PM used in this experiment, SRM-1648a, contains heavy metals, such as copper, lead, nickel, and manganese, and is composed of particles varying in size from 0.1 to 100 μm. Therefore, it was not possible to determine which chemical component or particle size class had the greatest effect on the hVFF. Further studies are thus required.

In addition, we showed that the cytotoxic effects of PM on hVFF were decreased by treatment with NAC, which is a ROS scavenger and antioxidant commonly used to reduce local inflammation by down-regulating the expression of inflammatory cytokines [47]. NAC has also been reported to reduce the expression of NF-κB, thereby regulating several other mechanisms of inflammation [48]. NAC is used clinically for the treatment of various inflammatory diseases, such as oral mucositis and dermatitis [49,50]. Thus, it may have potential as a therapeutic agent to prevent or treat the inflammatory response of the VF to PM. Further animal studies are required to validate our results.

In conclusion, this study showed that PM induces an inflammatory response in hVFF via the expression of pro-inflammatory cytokines in the MAPK/NF-κB signaling pathway. These mechanisms were activated by PM-induced ROS; the inflammatory response promoted the differentiation of hVFF into myofibroblasts, thereby promoting fibrosis of the VF. These results increase our understanding on the impact of PM on the larynx, which was not well-understood previously, and could inform therapeutic modalities for reducing the effects of PM on VF.

## 4. Materials and Methods

### 4.1. Epidemiological Data Acquisition

From January 2016 to December 2018, publicly available monthly air quality data provided by the Korea Environment Corporation were analyzed [51]. Only PM 10/PM 2.5 concentration data for Seoul, Republic of Korea, were extracted and analyzed. Disease prevalence data were acquired from the Healthcare Bigdata Hub of the Health Insurance Review and Assessment Service [52]. From January 2016 to December 2018, data for patients diagnosed with acute laryngitis/tracheitis (Korean Standard Classification of Diseases (KCD) code J04) at clinics in Seoul were extracted and analyzed.

### 4.2. Cell Culture

hVFF were isolated from tissues collected from normal human VF (contralateral, uninvolved VF of a glottic cancer patient who underwent total laryngectomy) and cultured, as described previously [53]. The Institutional Review Board of Chungnam National University Hospital approved this study (CNUH 2020-03-013). All the methods were performed in accordance with the Institutional Review Board of Chungnam National University Hospital guideline and regulation. The study participants provided an informed consent form before participating. Briefly, the cells were cultured with 10% fetal bovine serum (FBS; Gibco, Grand Island, NY, USA), 100 U/mL of penicillin-streptomycin (Gibco), nonessential amino acids (NEAA; Sigma-Aldrich, St. Louis, MO, USA), and 0.05% trypsin-EDTA (Gibco) in high-glucose Dulbecco’s modified Eagle’s medium (h-DMEM; Gibco) at 37 °C and 5% CO_2_. hVFF were used in the experiment after the 5th–10th passage.

### 4.3. Cell Proliferation/Counting Assay

Isolated hVFF were seeded in 96-well plates, at a density of 5 × 10^3^ cells per well, in h-DMEM medium containing 10% FBS. The PM used was from the National Institute of Standards and Technology (SRM-1648a; NIST, Gaithersburg, MD, USA). The particle size and composition are specified in the certificate [54]. Before PM treatment, sonification was performed for about 1 h to prevent the aggregation of particles. After treatment with PM, the hVFF cell viability was measured using the cell proliferation reagent, WST-1 (Roche Diagnostics, Indianapolis, IN, USA). WST-1 formazan was quantitated at 450 nm using an enzyme-linked immunosorbent assay reader. The results are presented as percentages relative to control cells. Cell counting assay was performed by LUNA-II™ Automated Cell Counter (Logos biosystems, Gyunggi, Korea).

### 4.4. Cell Cytotoxic Assay

Isolated hVFF were seeded in 96-well plates, at a density of 5 × 10^3^ cells per well, in h-DMEM medium containing 10% FBS. After treatment with PM, the cells were trypsinized and stained after being diluted 1:1 with 0.4% trypan blue dye. The stained cells were counted using a LUNA-II™ Automated Cell Counter (Logos biosystems, Gyunggi, Korea). The results are presented as percentages relative to control cells.

### 4.5. mRNA Isolation and Quantitative Real-Time Reverse Transcriptase Polymerase Chain Reaction (RT-PCR)

Total RNA was isolated using TRIzol (Invitrogen, Life Technologies, Carlsbad, CA, USA). Complementary DNA (cDNA) was prepared from the total RNA using M-MLV reverse transcriptase and oligo-dT primers (Invitrogen). RT-PCR was performed using cDNA, QuantiTect SYBR Green PCR Master Mix (Qiagen, Hilden, Germany), and specific primers for IL-6, IL-1β, and glyceraldehyde-3-phosphate dehydrogenase (GAPDH). The PCR reactions were performed for 40 cycles of 95 °C for 15 s, 60 °C for 1 min, and 72 °C for 1 min. The primer sequences were as follows: IL-6-F: 5′- TACCCCCAGGAGAAGATTCC -3′/IL-6-R: 5′- TTTTCTGCCAGTGCCTCTTT -3′, IL-1β-F: 5′-GTGGCAATGAGGATGACTTGTTC-3′/IL-1β-R: 5′-TAGTGGTGGTCGGAGATTCGTA-3′ and GAPDH-F: 5′-ACC CAG AAG ACT GTG GAT GG-3′/GAPDH-R:F:5′-TTC TAG ACG GCA GGT CAG GT-3′. Samples were analyzed in triplicate, and GAPDH was used as an endogenous control.

### 4.6. Western Blot Analysis

The cells were lysed in buffer containing 150 mM of NaCl, 1.0% Nonidet-P 40, 0.5% sodium deoxycholate, 0.1% sodium dodecyl sulfate, 50 mM of Tris, at pH 8.0, and a protease inhibitor cocktail (Roche Applied Science, Penzberg, Germany). Proteins were electrophoresed on 10% polyacrylamide gels and transferred to polyvinylidene fluoride membrane. Next, the transferred membrane was blocked with 5% skim milk. The following primary antibodies were used for Western blot analysis: anti-p-P65 (S536), anti-total P65, anti-p-IκBα, anti-total IκBα, anti-p-ERK(T202/Y204), anti-total-ERK, anti-p-JNK(T183/Y185), anti-total JNK, anti-p-P38(T180/Y182), anti-total P38, anti-β-actin (1:1,000; Cell Signaling Technology, Beverly, MA, USA), anti-fibronectin, anti- Col1A1, and anti-α-SMA (1:1000; Santa Cruz Biotechnology, Santa Cruz, CA, USA). Following incubation with the corresponding horseradish peroxidase-conjugated secondary antibodies (1:1,000; Santa Cruz Biotechnology), immunoreactive bands were visualized by enhanced chemiluminescence (ECL; Bio-Rad Laboratories, Hercules, CA, USA). Nuclear cytoplasmic fractionation was performed by NE-PER R Nuclear and cytoplasmic extraction reagents (Thermo scientific) according to the manufacturer’s protocol. Cells were seeded in a 60 mm dish at a density of 5 × 10^5^ cells/well. After the PM treatment, the cells were washed with PBS, then lysed in cytoplasmic extraction reagent I and II (CERI and II) after centrifugation at ~16,000× *g* for 5 min. The supernatant (cytoplasmic fraction) was transferred to a new tube. The pellet was lysed in a cold nuclear extraction reagent (NER) for 40 min. The samples were rotated at ~16 000 (4 °C) for 10 min, and the supernatant was transferred to a new tube (nuclei). Lamin B and α-tubulin (Santa Cruz Biotechnology, Santa Cruz, CA, USA) were used as nuclear and cytoplasmic markers, respectively. The inhibitors used in Western blot analysis were as follows: Bay 11-7082 (NF-κB inhibitor), PD98059 (ERK inhibitor), SP600125 (JNK inhibitor), SB203580 (P38 inhibitor), and NAC (antioxidant/free radical scavenger) (Sigma-Aldrich). The Western blotting results were quantified using the Image J software (version 1.53a; National Institutes of Health, Betheshda, MD, USA), and the densitometry results were indicated by numbers in Western blotting.

### 4.7. Analysis of ROS Production

The general oxidative stress in cells was assessed using 2′,7′-dichlorodihydrofluorescein diacetate (H2DCFDA, Cat. #D399; Thermo Fisher Scientific, Waltham, MA, USA). Mitochondrial superoxide was assessed using MitoSOX (Cat. #D1168; Thermo Fisher Scientific). The cells were treated with PM and stained with either H2DCFDA (10 µM) or MitoSOX (5 µM) in Hank’s balanced salt solution (HBSS; Gibco) for 0.5 h. The cells were then collected and analyzed for ROS using flow cytometry (FACSCanto™; BD Biosciences, San Jose, CA, USA).

### 4.8. Animal Model

Nine healthy male Sprague-Dawley rats aged 8–10 weeks and weighing about 200–250 g were used in the experiment. The rats were acclimated to 21 ± 1 °C, 50 ± 5% humidity, and 12-h light/dark cycle conditions for 7 days, with free access to food and water. The experimental procedure was approved by the Chungnam University School of Medicine Animal Experiment Ethics Committee (202003A-CNU-063). To avoid irritation of the VF, anesthesia was performed via intramuscular injection of Zoletil (10 mg/kg; Virbac, Carros, France). Nine rats were randomly divided into a control group consisting of three animals and an experimental group consisting of six animals. In the experimental group, 1 mL of PM diluted in 1 mg/mL distilled water was sprayed through the oral cavity five times every 3 days, using a throat spraying device designed for the tracheal intubation of small animals (#5380; MILA International, Florence, KY, USA). The control group animals were injected with 1 mL of normal saline (0.9% NaCl) using the same method. Three rats in the experimental group were sacrificed 20 days after the start of the experiment. The remaining rats in the experimental and control groups were sacrificed after 50 days.

### 4.9. Histological Analysis

The part of the larynx containing the VF was excised and the specimen was fixed in 4% neutral buffered formalin solution (Sigma-Aldrich). The false VF and subglottic level tissue were separated under microscope observation, and only glottic-level tissue was prepared (Figure 6B). After preparing paraffin samples via standard paraffin embedding procedures, the specimen was sliced (slice thickness = 6 μm). H&E, Masson’s trichrome, and Alcian blue staining were performed, after which histopathological changes were observed and analyzed using an optical microscope.

### 4.10. Immunohistochemical Analysis

After preparing the slides, three slides from each specimen were selected randomly. For immunohistochemical analysis, tissue were stained with anti-IL-6 antibody (1:800; Abcam, Cambridge, UK), p-NF-κB antibody (1:200; Cell Signaling Technology), and anti-Col1A1 antibody (1: 100; Santa Cruz Biotechnology). After washing three times with phosphate-buffered saline (PBS), the tissues were stained with secondary antibodies according to the manufacturer’s instructions and incubated overnight at room temperature (GBI Labs, Bothell, WA, USA). The images were analyzed using an optical microscope. Three points were randomly selected from each slide and quantified using the Image J software (National Institutes of Health, Betheshda, MD, USA).

### 4.11. Statistical Analysis

The correlation between the airborne PM 10/2.5 concentration and the incidence of acute laryngitis/tracheitis was analyzed using Pearson’s correlation. The coefficient of correlation (r) has a value between −1 and 1, where a positive value indicates a positive correlation and vice versa. The coefficient of determination (r2) indicates the proportion of variance in the dependent variable that is predictable from the independent variables. P-values of less than 5% were considered statistically significant. All in vitro experiments were repeated three times, and the data were analyzed using the two-tailed Student’s t-test or one-way analysis of variance followed by Tukey’s post hoc test. Data are expressed as means ± SD. * *p* < 0.05, ** *p* < 0.01, *** *p* < 0.001. SPSS statistical software for Windows (version 20.0; SPSS Inc., Chicago, IL, USA) was used for all statistical analyses.

## Supplementary Materials

Supplementary materials can be found at https://www.mdpi.com/1422-0067/21/18/6643/s1.

## Abbreviations

PM	Particulate matter
VF	Vocal folds
hVFF	Human vocal fold fibroblasts
NF-κB	Nuclear factor-κB
mRNA	Messenger RNA
IL	Interleukin
IκBα	Nuclear factor of kappa light polypeptide gene enhancer in B-cells inhibitor alpha
MAPK	Mitogen-activated protein kinase
ROS	Reactive oxygen species
ERK	Extracellular signal-regulated kinase
JNK	c-Jun N-terminal kinase
NAC	N-acetylcysteine
ECM	Extracellular matrix
TGF- β1	Transforming growth factor β1
Col1A1	Alpha-1 type I collagen
α-SMA	α-smooth muscle actin
H&E	Hematoxylin and eosin
RT-PCR	Quantitative real-time reverse transcriptase polymerase chain reaction
cDNA	Complementary DNA
GAPDH	Glyceraldehyde-3-phosphate dehydrogenase

## References

[1] Huang Y.-C.T. (2014). Outdoor Air Pollution. J. Occup. Environ. Med..

[2] Guarnieri M., Balmes J.R. (2014). Outdoor air pollution and asthma. Lancet.

[3] Liu S., Zhou Y., Liu S., Chen X., Zou W., Zhao D., Li X., Pu J., Huang L., Chen J. (2016). Association between exposure to ambient particulate matter and chronic obstructive pulmonary disease: Results from a cross-sectional study in China. Thorax.

[4] Weichenthal S., Lavigne E., Evans G.J., Pollitt K.J.G., Burnett R.T. (2016). Fine Particulate Matter and Emergency Room Visits for Respiratory Illness. Effect Modification by Oxidative Potential. Am. J. Respir. Crit. Care Med..

[5] Fajersztajn L., Veras M.M., Barrozo L., Saldiva P.H.N. (2013). Air pollution: A potentially modifiable risk factor for lung cancer. Nat. Rev. Cancer.

[6] Lee D.C., Choi H., Oh J.-M., Hong Y., Jeong S.H., Kim C.-S., Kim D.-K., Cho W.-K., Kim S.W., Kim S.W. (2018). The effect of urban particulate matter on cultured human nasal fibroblasts. Int. Forum Allergy Rhinol..

[7] Fujii T., Hayashi S., Hogg J.C., Vincent R., Van Eeden S.F. (2001). Particulate matter induces cytokine expression in human bronchial epithelial cells. Am. J. Respir. Cell Mol. Boil..

[8] De Grove K.C., Provoost S., Brusselle G.G., Joos G.F., Maes T. (2018). Insights in particulate matter-induced allergic airway inflammation: Focus on the epithelium. Clin. Exp. Allergy.

[9] Roy N., Merrill R.M., Gray S.D., Smith E.M. (2005). Voice Disorders in the General Population: Prevalence, Risk Factors, and Occupational Impact. Laryngoscope.

[10] Bhattacharyya N. (2014). The prevalence of voice problems among adults in the United States. Laryngoscope.

[11] Cohen S.M., Kim J., Roy N., Asche C., Courey M. (2012). Direct health care costs of laryngeal diseases and disorders. Laryngoscope.

[12] Tateya I., Tateya T., Sohn J.-H., Bless D.M. (2016). Histological Effect of Basic Fibroblast Growth Factor on Chronic Vocal Fold Scarring in a Rat Model. Clin. Exp. Otorhinolaryngol..

[13] Hirano S., Heisey D., Bless D.M., Ford C.N. (2003). Effect of growth factors on hyaluronan production by canine vocal fold fibroblasts. Ann. Otol. Rhinol. Laryngol..

[14] Hiwatashi N., Bing R., Kraja I., Branski R.C. (2017). Mesenchymal stem cells have antifibrotic effects on transforming growth factor-beta1-stimulated vocal fold fibroblasts. Laryngoscope.

[15] Øvrevik J., Refsnes M., Låg M., Holme J.A., Schwarze P.E. (2015). Activation of Proinflammatory Responses in Cells of the Airway Mucosa by Particulate Matter: Oxidant- and Non-Oxidant-Mediated Triggering Mechanisms. Biomolecules.

[16] Viatour P., Merville M.-P., Bours V., Chariot A. (2005). Phosphorylation of NF-κB and IκB proteins: Implications in cancer and inflammation. Trends Biochem. Sci..

[17] Wang J., Huang J., Wang L., Chen C., Yang D., Jin M., Bai C., Song Y. (2017). Urban particulate matter triggers lung inflammation via the ROS-MAPK-NF-kappaB signaling pathway. J. Thorac. Dis..

[18] Yan Z., Jin Y., An Z., Liu Y., Samet J.M., Wu W. (2016). Inflammatory cell signaling following exposures to particulate matter and ozone. Biochim. Biophys. Acta (BBA)—Gen. Subj..

[19] Limón-Pacheco J., Gonsebatt M.E. (2009). The role of antioxidants and antioxidant-related enzymes in protective responses to environmentally induced oxidative stress. Mutat. Res. Toxicol. Environ. Mutagen..

[20] Dhouib I.E., Jallouli M., Annabi A., Gharbi N., Elfazaa S., Lasram M.M. (2016). A minireview on N -acetylcysteine: An old drug with new approaches. Life Sci..

[21] Border W.A., Noble N.A. (1994). Transforming growth factor beta in tissue fibrosis. N. Engl. J. Med..

[22] Won H.-R., Song E.H., Won J.E., Lee H.Y., Kang S.U., Shin Y.S., Kim C.-H. (2019). Liquid-type non-thermal atmospheric plasma ameliorates vocal fold scarring by modulating vocal fold fibroblast. Exp. Boil. Med..

[23] Lim X., Tateya I., Tateya T., Muñoz-Del-Río A., Bless D.M. (2006). Immediate inflammatory response and scar formation in wounded vocal folds. Ann. Otol. Rhinol. Laryngol..

[24] Brook R.D., Franklin B., Cascio W., Hong Y., Howard G., Lipsett M., Luepker R., Mittleman M., Samet J., Smith S.C. (2004). Air Pollution and Cardiovascular Disease. Circulation.

[25] Kelly F.J., Fussell J.C. (2011). Air pollution and airway disease. Clin. Exp. Allergy.

[26] Kim K.-H., Kabir E., Kabir S. (2015). A review on the human health impact of airborne particulate matter. Environ. Int..

[27] Shah A.S.V., Langrish J.P., Nair H., McAllister D.A., Hunter A.L., Donaldson K., Newby D.E., Mills N.L. (2013). Global association of air pollution and heart failure: A systematic review and meta-analysis. Lancet.

[28] Girguis M.S., Strickland M.J., Hu X., Liu Y., Bartell S., Vieira V.M. (2016). Maternal exposure to traffic-related air pollution and birth defects in Massachusetts. Environ. Res..

[29] Calderón-Garcidueñas L., Solt A.C., Henríquez-Roldán C., Torres-Jardon R., Nuse B., Herritt L., Villarreal-Calderón R., Osnaya N., Stone I., García R. (2008). Long-term Air Pollution Exposure Is Associated with Neuroinflammation, an Altered Innate Immune Response, Disruption of the Blood-Brain Barrier, Ultrafine Particulate Deposition, and Accumulation of Amyloid β-42 and α-Synuclein in Children and Young Adults. Toxicol. Pathol..

[30] Sun Q., Yue P., Deiuliis J.A., Lumeng C.N., Kampfrath T., Mikolaj M.B., Cai Y., Ostrowski M., Lu B., Parthasarathy S. (2009). Ambient air pollution exaggerates adipose inflammation and insulin resistance in a mouse model of diet-induced obesity. Circulation.

[31] Sundaresan A.S., Hirsch A.G., Storm M., Tan B.K., Kennedy T.L., Greene J.S., Kern R.C., Schwartz B.S. (2015). Occupational and environmental risk factors for chronic rhinosinusitis: A systematic review. Int. Forum Allergy Rhinol..

[32] Tang W., Du L., Sun W., Yu Z., He F., Chen J., Li X., Li X., Yu L., Chen D. (2017). Maternal exposure to fine particulate air pollution induces epithelial-to-mesenchymal transition resulting in postnatal pulmonary dysfunction mediated by transforming growth factor-β/Smad3 signaling. Toxicol. Lett..

[33] Thevenot P.T., Saravia J.S., Jin N., Giaimo J.D., Chustz R.E., Mahne S., Kelley M.A., Hebert V.Y., Dellinger B., Dugas T.R. (2013). Radical-Containing Ultrafine Particulate Matter Initiates Epithelial-to-Mesenchymal Transitions in Airway Epithelial Cells. Am. J. Respir. Cell Mol. Boil..

[34] Shvedova A.A., Pietroiusti A., Fadeel B., Kagan V.E. (2012). Mechanisms of carbon nanotube-induced toxicity: Focus on oxidative stress. Toxicol. Appl. Pharmacol..

[35] Øvrevik J., Refsnes M., Låg M., Brinchmann B., Schwarze P.E., Holme J.A. (2017). Triggering Mechanisms and Inflammatory Effects of Combustion Exhaust Particles with Implication for Carcinogenesis. Basic Clin. Pharmacol. Toxicol..

[36] Hamad S.H., Schauer J.J., Antkiewicz D.S., Shafer M.M., Kadhim A.K. (2016). ROS production and gene expression in alveolar macrophages exposed to PM2.5 from Baghdad, Iraq: Seasonal trends and impact of chemical composition. Sci. Total. Environ..

[37] Baker J., Orr B., Holl M.B. (2007). Nanoparticle Interactions with Biological Membranes. Nanotoxicology.

[38] Beamer C.A., Holian A. (2008). Silica suppresses Toll-like receptor ligand-induced dendritic cell activation. FASEB J..

[39] Rui W., Guan L., Zhang F., Zhang W., Ding W. (2015). PM 2.5 -induced oxidative stress increases adhesion molecules expression in human endothelial cells through the ERK/AKT/NF-?B-dependent pathway. J. Appl. Toxicol..

[40] Wang T., Chiang I.T., Moreno-Vinasco L., Lang G.D., Pendyala S., Samet J.M., Geyh A.S., Breysse P.N., Chillrud S.N., Natarajan V. (2009). Particulate Matter Disrupts Human Lung Endothelial Barrier Integrity via ROS- and p38 MAPK–Dependent Pathways. Am. J. Respir. Cell Mol. Boil..

[41] Wang R., Xiao X., Shen Z., Cao L., Cao Y. (2016). Airborne fine particulate matter causes murine bronchial hyperreactivity via MAPK pathway-mediated M3muscarinic receptor upregulation. Environ. Toxicol..

[42] Hanson S.E., Kim J., Dds B.H.Q.J., Bradley B., Breunig M.J., Hematti P., Thibeault S.L. (2010). Characterization of mesenchymal stem cells from human vocal fold fibroblasts. Laryngoscope.

[43] Ban M.J., Lee S.C., Park J.H., Park K.N., Kim H.K., Lee S.W. (2020). Regenerative Efficacy of Fibroblast Growth Factor for the Treatment of Aged Vocal Fold: From Animal Model to Clinical Application. Clin. Otolaryngol..

[44] Bai Y., Sun Q. (2016). Fine particulate matter air pollution and atherosclerosis: Mechanistic insights. Biochim. Biophys. Acta (BBA)—Gen. Subj..

[45] Loxham M. (2014). Harmful effects of particulate air pollution: Identifying the culprits. Respirology.

[46] Park M., Lee J.S., Park M.K. (2019). The Effects of Air Pollutants on the Prevalence of Common Ear, Nose, and Throat Diseases in South Korea: A National Population-Based Study. Clin. Exp. Otorhinolaryngol..

[47] Aihara M., Dobashi K., Akiyama M., Naruse I., Nakazawa T., Mori M. (2000). Effects of N-acetylcysteine and ambroxol on the production of IL-12 and IL-10 in human alveolar macrophages. Respiration.

[48] He D., Behar S., Roberts J.E., Lim H.W. (1996). The effect of L-cysteine and N-acetylcysteine on porphyrin/heme biosynthetic pathway in cells treated with 5-aminolevulinic acid and exposed to radiation. Photodermatol. Photoimmunol. Photomed..

[49] Sio T.T., Blanchard M.J., Novotny P.J., Patel S.H., Rwigema J.-C.M., Pederson L.D., McGee L.A., Gamez M.E., Seeger G.R., Martenson J.A. (2019). N-Acetylcysteine Rinse for Thick Secretion and Mucositis of Head and Neck Chemoradiotherapy (Alliance MC13C2). Mayo Clin. Proc..

[50] Demirel C., Kilciksiz S., Evirgen-Ayhan S., Gurgul S., Erdal N. (2010). The preventive effect of N-acetylcysteine on radiation-induced dermatitis in a rat model. J. B.U.ON. Off. J. Balk. Union Oncol..

[51] Air Korea. https://www.airkorea.or.kr/eng.

[52] Healthcare Bigdata Hub. https://opendata.hira.or.kr/home.do.

[53] Thibeault S.L., Li W., Bartley S. (2008). A method for identification of vocal fold lamina propria fibroblasts in culture. Otolaryngol. Neck Surg..

[54] https://www-s.nist.gov/srmors/view_cert.cfm?srm=1648A.

