# Supplementary material for: Effect of Urban Particulate Matter on Vocal Fold Fibrosis through the MAPK/NF-κB Signaling Pathway
Source: Int J Mol Sci. 2020 Sep 10;21(18):6643. doi: 10.3390/ijms21186643 (PMC7555340; doi:10.3390/ijms21186643)
Supplement: Supplementary file 1 [file ijms-21-06643-s001.pdf]

International Journal of
Molecular Sciences

MDPI

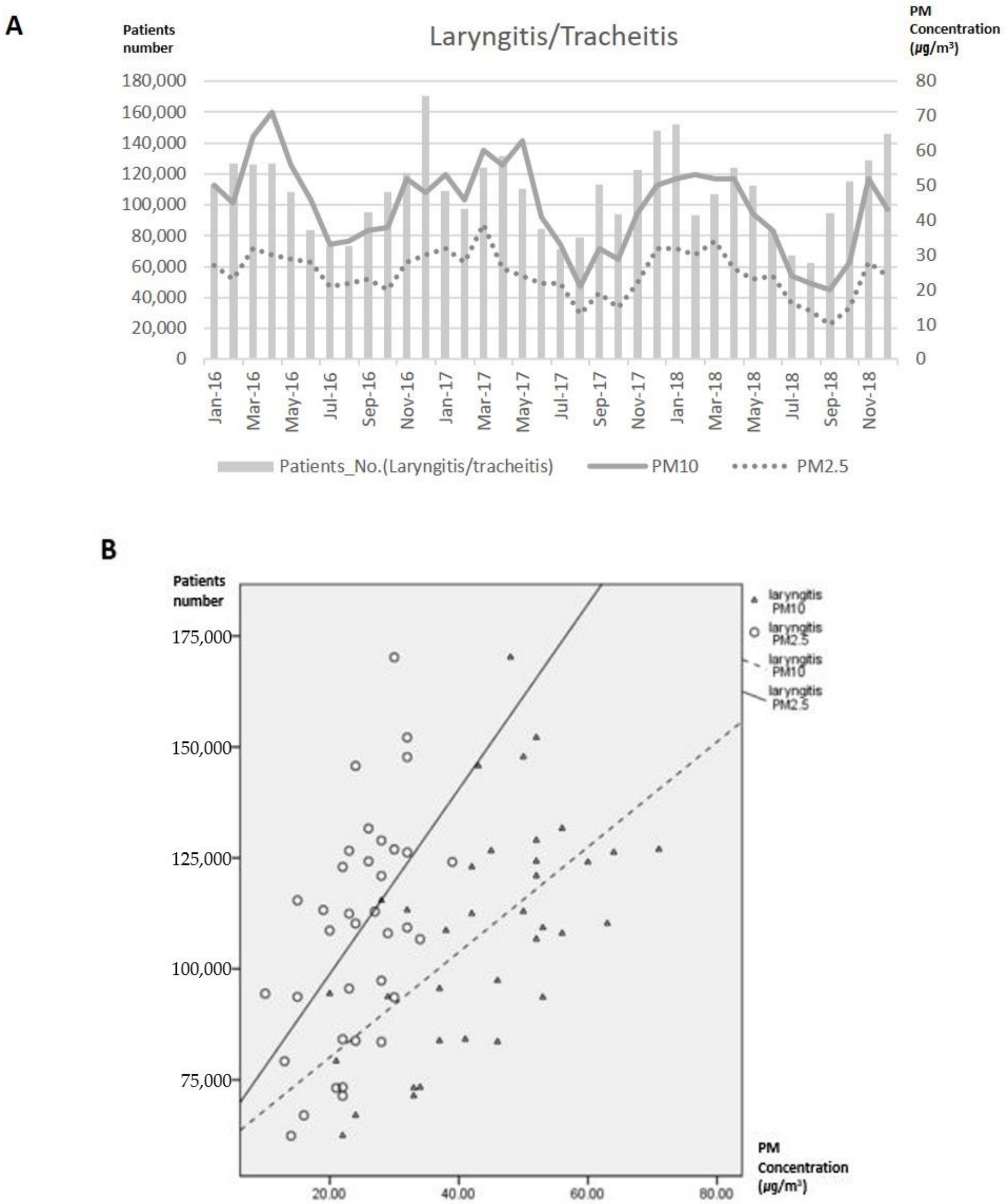

		PM 10	PM 2.5
Acute Laryngitis /tracheitis	r	0.593	0.533
	r^2	0.353	0.284
	P-value	<0.001***	<0.001***

r : coefficient of correlation
r^2 : coefficient of determination

Figure S1. Relationship between the incidence of acute laryngitis/tracheitis and the PM 10/PM 2.5 concentration. (**A**) Graph of PM concentration and number of acute laryngitis/tracheitis patients in Seoul, Republic of Korea from January 2016 to December 2018. (**B**) Results of Pearson correlation coefficient analysis. The airborne PM 10/PM 2.5 concentration and incidence of acute laryngitis/tracheitis were significantly positively correlated. r: coefficient of correlation, r2: coefficient of determination. *** $p < 0.001$.
